# Reproductive factors related to the risk of colorectal cancer by subsite: a case-control analysis

**DOI:** 10.1038/sj.bjc.6690302

**Published:** 1999-04

**Authors:** K-Y Yoo, K Tajima, M Inoue, T Takezaki, K Hirose, N Hamajima, S K Park, D H Kang, T Kato, T Hirai

**Affiliations:** Department of Preventive Medicine, Seoul National University College of Medicine, 28 Yongon-dong, Seoul, Chongno-gu, 110-799, Korea; Division of Epidemiology, Aichi Cancer Center Research Institute; Department of Gastroenterological Surgery, Aichi Cancer Center Hospital, 1-1 Kanokoden, Chikusa-ku, Nagoya, 464, Japan

**Keywords:** colon neoplasms, reproduction, risk factors

## Abstract

The authors hypothesized that reproductive factors of colorectal cancer, which are probably mediated by endogenous hormones, would differ according to colonic subsite. Information on reproductive factors was obtained from 372 female colorectal cancer cases (113 proximal colon, 126 distal colon, 133 rectum) and 31 061 cancer-free controls at the Aichi Cancer Center Hospital, Japan, between 1988 and 1995. Multiple logistic analysis showed that late age at interview, family history of colorectal cancer among first-degree relatives, menstrual regularity, late age at menopause, late age at first pregnancy and late age at first full-term pregnancy were significantly associated with the risk of colorectal cancer. None of the risk factors were significantly dissociated between colon and rectal cancer. In polytomous logistic regression analysis, particularly noteworthy was the fact that the odds ratios for age at menarche (*P*-value for heterogeneity of odds ratios = 0.010), age at first pregnancy (*P* = 0.016) and age at first full-term pregnancy (*P* = 0.028) were significantly higher for distal than for proximal colon cancer. This study supports the hypotheses that there might be an association between reproductive factors and risk of colon cancer, and that the carcinogenesis of colon cancer, by subsite, might show aetiologic distinctions. © 1999 Cancer Research Campaign


*British Journal of Cancer* (1999) **79**(11/12), 1901–1906
© 1999 Cancer Research Campaign
Article no. bjoc.1998.0302

### Reproductive factors related to the risk of colorectal cancer by subsite: a case-control analysis

**K-Y Yoo[1], K Tajima[2], M Inoue[2], T Takezaki[2], K Hirose[2], N Hamajima[2], SK Park[1], DH Kang[1], T Kato[3] and T Hirai[3]**

[1]Department of Preventive Medicine, Seoul National University College of Medicine, 28 Yongon-dong, Chongno-gu, Seoul 110-799, Korea; [2]Division of Epidemiology, Aichi Cancer Center Research Institute; [3]Department of Gastroenterological Surgery, Aichi Cancer Center Hospital, 1-1 Kanokoden, Chikusa-ku, Nagoya 464, Japan

**Summary** The authors hypothesized that reproductive factors of colorectal cancer, which are probably mediated by endogenous hormones, would differ according to colonic subsite. Information on reproductive factors was obtained from 372 female colorectal cancer cases (113 proximal colon, 126 distal colon, 133 rectum) and 31 061 cancer-free controls at the Aichi Cancer Center Hospital, Japan, between 1988 and 1995. Multiple logistic analysis showed that late age at interview, family history of colorectal cancer among first-degree relatives, menstrual regularity, late age at menopause, late age at first pregnancy and late age at first full-term pregnancy were significantly associated with the risk of colorectal cancer. None of the risk factors were significantly dissociated between colon and rectal cancer. In polytomous logistic regression analysis, particularly noteworthy was the fact that the odds ratios for age at menarche ($P$-value for heterogeneity of odds ratios = 0.010), age at first pregnancy ($P = 0.016$) and age at first full-term pregnancy ($P = 0.028$) were significantly higher for distal than for proximal colon cancer. This study supports the hypotheses that there might be an association between reproductive factors and risk of colon cancer, and that the carcinogenesis of colon cancer, by subsite, might show aetiologic distinctions.

**Keywords:** colon neoplasms; reproduction; risk factors

One of the most dramatic features of colorectal cancer is that there is a large difference in incidence rates between highly westernized and non-westernized countries. The initial suggestion that environmental factors were important determinants of colorectal cancer risk was based on epidemiologic studies showing marked variations in colorectal cancer death rates in different parts of the world (Armstrong et al, 1975). As is true with stomach cancer, differences in diet are thought to be a major underlying factor in the different incidence rates of colorectal cancer (Zaridze, 1983). Based on the fat and fibre hypothesis, a diet high in meat, fat and protein, and low in dietary fibre is considered to be important in the aetiology of colon cancer (Potter et al, 1993).

Since the first observation that, among nuns, the incidence not only of known hormonal cancers but also of colon cancer is disproportionate (Fraumeni et al, 1969), a variety of evidence has shown that reproductive factors may alter the risk of colon cancer in women (Weiss et al, 1981; Kune et al, 1989; Bostick et al, 1994; Martinez et al, 1997). Moreover, the results of other studies have suggested that there might be differences in the aetiology of colorectal cancer by subsite. The pattern of international variation in the incidence of colon cancer shows that, in countries with higher rates, excess tumours are found largely in the distal colon (Correa and Haenszel, 1978). It has been reported that the incidence of cancer of the caecum and ascending colon is 10–20% higher in women than in men at all ages (McMichael and Potter, 1980, 1983). There are clear physiologic distinctions between the proximal and distal colon, i.e. undigested dietary fibre undergoes prolonged fermentation and degradation in the proximal colon; faecal transit is thought to be much slower against gravity, proximally (Robbins 1974; Cummings, 1983). However, reports examining reproductive factors in colorectal cancer patients, particularly by subsite, have not produced clear and consistent findings (Potter and McMichael, 1983; Howe et al, 1985; Peters et al, 1990; Kvale and Heuch, 1991; de Verdier and London, 1992; Cantor et al, 1993; Kravdal et al, 1993; Jacobs et al, 1994; Slattery et al, 1995; Kampman et al, 1997). Incidence and mortality for female colon cancer have been increasing in some Asian countries, i.e. Korea and Japan. However, no epidemiologic studies on reproductive factors have been conducted in the countries, except for a case-control study of dietary factors by colonic subsite (Inoue et al, 1995).

This study, conducted through a hospital-based case-control study in Japan, aimed both to assess the relationship between female reproductive factors and the risk of colorectal cancer, and to provide an evidence that risk factors may differ according to colonic subsite.

### MATERIALS AND METHODS

### Study subjects

General characteristics of the study population and data collection procedures have been described elsewhere (Yoo et al, 1992, 1997; Hirose et al, 1995). Briefly, a self-administered questionnaire concerning lifestyle, reproductive history and other factors was given routinely to first-visit outpatient adults at the Aichi Cancer Center Hospital, Nagoya, Japan. Most outpatients visited the

*Received 28 April 1998*
*Revised 18 July 1998*
*Accepted 20 July 1998*

*Correspondence to:* K-Y Yoo, Department of Preventive Medicine, Seoul National University College of Medicine, 28 Yongon-dong, Chongno-gu, Seoul 110-799, Korea

**Table 1** Number of cases by subsite identified by pathologic findings among 372 colorectal cancer patients interviewed at Aichi Cancer Center Hospital, Japan, 1988–1995

| Colonic sites | Subsites | No. of cases |
|---|---|---|
| Proximal colon | Hepatic flexure | 53 |
| | Caecum | 11 |
| | Appendix | 1 |
| | Ascending colon | 31 |
| | Transverse colon | 17 |
| Distal colon | Descending colon | 9 |
| | Sigmoid colon | 117 |
| Rectum | | 133 |
| Total | | 372 |

hospital for the detection or diagnosis of cancer, not for treatment. The present report is based on women who completed the questionnaire between January 1988 and December 1995.

Cases were incident colorectal cancer patients who had undergone surgery and about whom subsite-specific information was collected, based on both clinical and histopathologic examination at the hospital. Of all the female cases ($n$ = 376), four women who had a past history of any malignancies other than colorectal cancer were excluded. Controls were all women confirmed by diagnostic procedures at the hospital to be cancer-free ($n$ = 32 416). Women younger than 20 ($n$ = 731) and those with a past history of cancer ($n$ = 612) were also excluded from the eligible population, as were 12 women with missing observations in at least three of four variables (age at menarche, age at menopause, age at first full-term pregnancy and average months of breast feeding per child). Included in this analysis were 372 colorectal cancer cases and 31 061 controls.

Based on surgical records, cases were divided into three subgroups according to anatomic site of the primary lesion: proximal colon (appendix, caecum, ascending and transverse colon), distal colon (descending and sigmoid colon) and rectum (recto-sigmoid colon, rectum). Included were 113 patients with proximal colon cancer, 126 with distal colon cancer and 133 with rectal cancer (Table 1).

### Data analysis

All the colorectal cases were compared to controls using the unconditional multiple logistic regression model. Odds ratios (OR) and 95% confidence intervals (CI) were calculated for known and suspected risk factors, which were simultaneously included in the model. The statistical significance of the difference between colon versus rectum, as well as between proximal versus distal colon, was determined by a case-to-case comparison, using the multiple logistic regression model (Breslow and Day, 1980; Fox, 1984). The PC-SAS and EGRET systems were used for statistical analysis (SAS Institute, 1987; Statistics and Epidemiology Research Corporation, 1990).

The following variables were included in each model as categorical variables: occupation (housewives vs all others), family history of colorectal cancer among first-degree relatives, menstruation at the age of between 20 and 29, menopausal status, full-term pregnancy history, alcohol drinking and cigarette smoking. Included in each model as continuous variables were age at interview, age at menarche, age at menopause, age at first pregnancy,

**Table 2** Adjusted risks of colorectal cancer among 372 cases and 31 061 controls interviewed at Aichi Cancer Center Hospital, Japan, 1988–1995

| Risk factors | Categories or units | All cases vs controls[a] aOR (95% CI) |
|---|---|---|
| Age at interview | per 5 years | 1.32 (1.23–1.42) |
| Occupation | Housewives | 1.0 |
| | All others | 0.88 (0.71–1.08) |
| Family history of colorectal cancer among first-degree relatives | No | 1.0 |
| | Yes | 2.58 (1.77–3.75) |
| Age at menarche | per 2 years | 1.08 (0.96–1.22) |
| Menstruation | Regular | 1.0 |
| | Irregular | 0.65 (0.49–0.86) |
| Age at menopause | per 5 years | 1.24 (1.02–1.50) |
| Age at first pregnancy[b] | per 5 years | 1.30 (1.11–1.51) |
| Number of pregnancies[b] | per unity | 0.98 (0.91–1.06) |
| History of full-term pregnancy | Nulliparous | 0.21 (0.08–0.58) |
| | Parous | |
| Age at first full-term pregnancy | per 5 years | 1.33 (1.13–1.56) |
| Number of full-term pregnancies | per unity | 1.07 (0.96–1.20) |
| Average months of breast feeding per child | per 3 months | 1.03 (0.99–1.08) |
| Alcohol drinking | Never | 1.0 |
| | Ever | 1.02 (0.78–1.32) |
| Cigarette smoking | Never | 1.0 |
| | Ever | 0.97 (0.71–1.33) |

[a]Relative risks were estimated as adjusted odds ratios (aOR) based on a case-control comparison. Odds ratios were adjusted for all covariates listed in the Table, in addition to menopausal status, ever had a full-term pregnancy, body mass index, weight at age 20, softness of faeces, and intake frequency of certain foods (rice, bean curd, fruits, ham/sausage) and their 95% confidence intervals were derived from regression coefficient and standard error in the linear logistic regression model. [b]Variables of pregnancy and full term pregnancy were not simultaneously included in the model, due to high colinearity.

number of pregnancies, age at first full-term pregnancy, number of full-term pregnancies and average months of breast feeding per child. Certain variables previously found to be significant (Inoue et al, 1995), e.g. softness of faeces (yes vs no) and intake frequency of certain foods (rice, bean curd, fruits, ham and sausage; frequently vs rarely), were incorporated into the model as dichotomous covariates. Due to high collinearity, variables of pregnancy (pregnancy history, age at first pregnancy, number of pregnancies) and variables of full-term pregnancy, (full-term pregnancy history, age at first full-term pregnancy, number of full-term pregnancies) were not simultaneously included in the model.

For continuous variables applying only to parous women, nulliparous women were assigned a value of zero. Because the variable ‘full-term pregnancy history’ was also included in the model, this removed the influence of nulliparity from the coefficient estimates for these continuous variables. Similarly, for the continuous variable ‘age at menopause’, premenopausal women were assigned a value of zero. For multivariate modelling, imputed missing values for continuous variables were replaced with age stratum-specific median values. Age at menarche for eight cases and 114 controls, age at menopause for eight controls, age at first pregnancy for two cases and 57 controls, number of pregnancies for no cases and 25 controls, age at first full-term pregnancy for two cases and 69 controls, number of full-term pregnancies for no cases and 48 controls, and the average months of breast feeding for three cases and 164 controls were imputed in this manner. For cases, missing

 *© Cancer Research Campaign 1999*

**Table 3** Results of multivariate logistic regression analysis to compare reproductive factors between proximal and distal colon cancer, Aichi Cancer Center Hospital, Japan, 1988–1995

| Risk factors | Categories or units | Proximal colon vs controls aOR (95% CI)[a] | Distal colon vs controls aOR (95% CI)[a] | *P*-value for intercase comparison[b] |
|---|---|---|---|---|
| No. of cases | | 113 | 126 | |
| No. of controls | | 31 061 | 31 061 | |
| Age at interview | per 5 years | 1.36 (1.20–1.54) | 1.22 (1.09–1.37) | 0.694 (1) |
| Occupation | Housewives | 1.0 | 1.0 | 0.823 (1) |
| | All others | 0.92 (0.63–1.34) | 0.80 (0.56–1.15) | |
| Family history of colon cancer[c] | No | 1.0 | 1.0 | 0.712 (1) |
| | Yes | 1.97 (0.95–4.11) | 3.02 (1.67–5.45) | |
| Age at menarche | per 2 years | 0.88 (0.70–1.10) | 1.34 (1.10–1.62) | 0.010 (1) |
| Menstruation | Regular | 1.0 | 1.0 | 0.442 (1) |
| | Irregular | 0.72 (0.44–1.20) | 0.53 (0.32–0.88) | |
| Age at menopause | per 5 years | 1.72 (1.20–2.48) | 1.09 (0.78–1.51) | 0.124 (1) |
| Age at first pregnancy[d] | per 5 years | 1.10 (0.84–1.45) | 1.63 (1.28–2.09) | 0.016 (1) |
| Number of pregnancies[d] | per unity | 0.95 (0.83–1.09) | 1.08 (0.96–1.22) | 0.202 (1) |
| Age at first full-term pregnancy | per 5 years | 1.17 (0.88–1.55) | 1.75 (1.36–2.25) | 0.028 (1) |
| Number of full-term pregnancies | per unity | 1.01 (0.83–1.23) | 1.19 (1.00–1.42) | 0.803 (1) |
| Average months of breast feeding per child | per 3 months | 0.99 (0.91–1.07) | 1.04 (0.97–1.13) | 0.403 (1) |
| Alcohol drinking | Never | 1.0 | 1.0 | 0.426 (1) |
| | Ever | 1.13 (0.71–1.80) | 0.78 (0.49–1.25) | |
| Cigarette smoking | Never | 1.0 | 1.0 | 0.944 (1) |
| | Ever | 0.64 (0.34–1.23) | 0.81 (0.46–1.42) | |

[a]Odds ratios were adjusted for all covariates (aOR) listed in the Table, in addition to menopausal status, ever had a full-term pregnancy, softness of faeces, intake frequency of certain foods (rice, bean curd, fruits, and ham/sausage), and their 95% confidence intervals were based on regression coefficients and standard errors of the multivariate logistic regression model. [b]Likelihood ratio test for difference between proximal and distal colon cancer cases, adjusted for all other covariates. ( ), degree of freedom. [c] Family history of colon cancer among first-degree relatives. [d] Variables of pregnancy and full-term pregnancy were not simultaneously included in the model, due to high collinearity.

values for menstruation between the age of 20 and 29 were considered to be irregular ($n$ = 4); for controls, missing values for this variable were considered to be regular ($n$ = 188). This conservative assumption biased the overall result for menstruation toward the null. Subjects with missing observations for alcohol drinking (34 controls) and cigarette smoking (one case and 31 controls) were considered to be never-users. A more detailed procedure has been described elsewhere (Yoo et al, 1997).

### RESULTS

Using a multiple logistic regression model, all cases were compared with controls. Table 2 shows that some risk factors are significantly related to the risk of colorectal cancer; these include age at interview (OR = 1.32 per 5 years; 95% CI = 1.23–1.42), family history of colorectal cancer among first-degree relatives (OR = 2.58; 95% CI = 1.77–3.75), menstruation at younger age (OR = 0.65; 95% CI = 0.49–0.86), age at menopause (OR = 1.24 per 5 years; 95% CI = 1.02–1.50), age at first pregnancy (OR = 1.30 per 5 years; 95% CI = 1.11–1.51) and age at first full-term pregnancy (OR = 1.33 per 5 years; 95% CI = 1.13–1.56). Occupation, age at menarche, number of pregnancies, number of full-term pregnancies, average months of breast feeding per child, alcohol drinking and cigarette smoking, however, were not significantly associated with the risk of colorectal cancer.

The results of multivariate comparison of risk factors between colon and rectal cancer, using a polytomous logistic regression model, shows that none of the variables previously found to be significant risk factors in colorectal cancer were significant. Accordingly, rectal cases were disregarded for further analysis, and the results are not presented.

The results of multiple polytomous logistic regression analysis to determine whether risk factors differed across the three subgroups (proximal colon cancer, distal colon cancer and controls) are shown in Table 3. Age at interview was positively associated with both proximal (OR = 1.36 per 5 years; 95% CI = 1.20–1.54) and distal colon cancer (OR = 1.22 per 5 years; 95% CI = 1.09–1.37), but heterogeneity of the odds ratios was not statistically significant ($P$ = 0.694). The odds ratio for family history of colon cancer among first-degree relatives was higher in distal (OR = 3.02; 95% CI = 1.67–5.45) than in proximal colon cancer (OR = 1.97; 95% CI = 0.95–4.11), but there was no significant difference in the odds ratios between proximal and distal colon cancer ($P$ = 0.712). Among the menstrual factors, age at menarche was negatively associated with proximal cancer (OR = 0.88 per 2 years; 95% CI = 0.70–1.10), but positively associated with distal colon cancer (OR = 1.34 per 2 years; 95% CI = 1.10–1.62) ($P$-value for heterogeneity of odds ratios = 0.010). However, menstrual regularity previously found significant in case-control comparison, did not show any significant difference in odds ratios between proximal and distal comparison ($P$-value for heterogeneity of odds ratios = 0.442). Although age at menopause was positively associated with proximal colon cancer (OR = 1.72 per 5 years; 95% CI = 1.20–2.48), the difference in odds ratios between this and distal colon cancer was not significant ($P$ = 0.124).

Particularly noteworthy was that apparent heterogeneity of odds ratios for reproductive factors of both pregnancy and full-term pregnancy between proximal and distal colon cancer was observed. The odds ratio for age at first pregnancy was significantly higher for distal (OR = 1.63 per 5 years; 95% CI = 1.28–2.09) than for proximal colon cancer (OR = 1.10 per 5 years; 95% CI = 0.84–1.45) ($P$-value for heterogeneity of odds ratios = 0.016). Similarly, the odds ratio for age at first full-term pregnancy was significantly higher for distal (OR =

*© Cancer Research Campaign 1999*

**Table 4** Summary of epidemiologic findings on the association between reproductive factors and colorectal cancer by subsite

| Authors (year) | Areas | Study design | Subjects | Subsites | OR[a] or RR[a] of colorectal cancer with increase in: Age at menarche | Age at menopause | No. of FTP[b] | Age at FFTP[c] |
|---|---|---|---|---|---|---|---|---|
| Potter and McMichael (1983) | Australia | Case-control | 155/311 | Colon | – | – | Dec | Inc |
| | | | | Rectum | – | – | Dec | Inc |
| | | | | Proximal | – | – | Dec | Inc |
| | | | | Distal | – | – | Dec | Inc |
| Howe et al (1985) | Toronto, Canada | Case-control | 229/242/257 | Colon | – | – | – | Inc[d] |
| | | | | Rectum | – | – | – | Inc[d] |
| | | | | Proximal | – | – | Inc | Inc |
| | | | | Distal | – | – | Dec | Inc |
| Peters et al (1990) | Los Angles County, USA | Case-control | 327/327 | Colon | U | U | Dec[d] | U |
| | | | | Proximal | U | Dec | Dec | U |
| | | | | Distal | U | U | Dec[d] | U |
| Kvale and Heuch (1991) | Norway | Cohort | 831/63090 | Colon | 0.87 | 1.11 | 0.93 | 1.16 |
| | | | | Rectum | 0.99 | 0.89 | 0.82 | 0.68 |
| | | | | Proximal | – | – | 0.69 | 0.64 |
| | | | | Distal | – | – | 1.19 | 1.25 |
| | | | | | | | 1.04 | 2.12[d] |
| de Verdier and London (1992) | Sweden | Case-control | 299/276 | Colon | 0.7 | 0.9 | Dec | U |
| | | | | Rectum | 0.8 | 1.0 | U | U |
| | | | | Proximal | 0.7 | 0.8 | U | Dec |
| | | | | Distal | 0.8 | 1.0 | U | Inc |
| Cantor et al (1993) | Iowa, USA | Case-control | 332/831 | Colon | – | – | U | U |
| | | | | Rectum | – | – | Inc | U |
| | | | | Proximal | – | – | 0.98 | – |
| | | | | Distal | – | – | 0.38[d] | – |
| Kravdal et al (1993) | Norway | Cohort | 859/1.1 million | Colon | – | – | U | Inc |
| | | | | Rectum | | | | |
| | | | | Proximal | – | – | Dec | U |
| | | | | Distal | – | – | Dec | Inc |
| Jacobs et al (1994) | Seattle, USA | Case-control | 193/194 | Colon | – | 1.13 | Dec | Dec[d] |
| | | | | Proximal | – | 0.84 | Dec | Dec |
| | | | | Distal | – | 1.39 | Dec | Dec |
| Slattery et al (1995) | Utah, USA | Cohort | 819/3202 | Colon | – | – | Dec | U |
| | | | | Proximal | – | – | 0.65[d] | U |
| | | | | Distal | – | – | U | U |
| Yoo et al (1997) | Japan | Case-control | 372/31 061 | Colon | 1.12 | 1.34[d] | 1.10 | 1.45[d] |
| | | | | Rectum | 1.02 | 1.08 | 1.04 | 1.10 |
| | | | | Proximal | 0.88 | 1.72[d] | 1.01 | 1.17 |
| | | | | Distal | 1.34[d] | 1.09 | 1.19[d] | 1.75[d] |

[a]Odds ratios and relative risks. [b]Full-term pregnancy. [c]First full-term pregnancy. [d]*P*-values less than 0.05. inc/dec/U : indicates increasing/decreasing/undefined trend in odds ratios or relative risks.

1.75 per 5 years; 95% CI = 1.36–2.25) than for proximal colon cancer (OR = 1.17 per 5 years; 95% CI = 0.88–1.55) (*P*-value for heterogeneity of odds ratios = 0.028). Neither the number of pregnancies nor the number of full-term pregnancies showed significant heterogeneity between proximal and distal colon cancer risk, however. The remaining variables, i.e. average months of breast feeding per child, alcohol drinking and cigarette smoking, showed no difference in odds ratios between proximal and distal colon cancer.

### DISCUSSION

Only a few studies have been conducted to determine whether menstrual factors are related to the risk of colon cancer. Table 4 shows that the overall picture from reports so far published is that there is no association between menstrual factors and colon or colorectal cancer (Peters et al, 1990; Kvale and Heuch, 1991; de Verdier and London, 1992; Jacobs et al, 1994; Kampman et al, 1997). Although we also failed to see the relationship between age at menarche and colorectal cancer, there is an apparent relationship of late age at menopause with significantly elevated risk of colon cancer, suggesting the possible role of menstrual factors in the aetiology of colorectal cancer.

A summary of nine studies on parity in relation to female colon cancer risk shows marked inconsistencies (Table 4). A community-based case-control study in Australia shows that increasing parity was associated with a decreasing risk of colon cancer (Potter and McMichael, 1983). A similar decreasing trend was observed in a population-based case-control study in Los Angeles, CA, USA (Peters et al, 1990) and among women diagnosed before age 65 in a cohort study in Utah, USA (Slattery et al, 1995). Cantor et al (1993) observed that parous women were at significantly decreased risk of colon cancer (OR = 0.67; 95% CI =

 *© Cancer Research Campaign 1999*

0.5–0.97), but no significant trend with parity was seen. Neither case-control study in Sweden (de Verdier and London, 1992) and in Seattle (Jacobs et al, 1994), nor two cohort studies in Norway (Kvale and Heuch, 1991; Kravdal et al, 1993) reported any significant evidence of a risk of colon or rectal cancer being influenced by parity, and this was consistent with the results in this study. The findings of the significant increasing trends in odds ratios with age at first full-term pregnancy observed in this study were consistent with the results of the earlier case-control studies (Potter and McMichael, 1983; Howe et al, 1985).

It is not yet clear whether colorectal cancers at different subsites represent aetiologically distinct forms of the disease and have different risk factor profiles. In previous studies, overall, there is no evidence that menstrual factors differ in their association with colon cancer risk by subsite (Table 4). In the present study, however, age at menarche was positively associated with distal colon cancer, although it did not show statistical significance in case-control comparison. Even though both menstrual regularity and late age at menopause were significantly associated with colorectal cancer risk, stratified analysis by subsite did not reveal a significant association. These findings should be pursued in further epidemiologic studies.

Reports on the dissociation between parity and colon cancer risk by subsite are quite inconsistent (Table 4). Two reports have found that parity only slightly decreases the risk of colon cancer in both sides of the colon, if at all (Potter and McMichael, 1983; Peters et al, 1990). In some studies, however, statistical testing – needed if trends in parity are to be revealed and subsequently described – was not carried out. Peters et al (1990) and Jacobs et al (1994), on the other hand, interpreted their case-control studies to indicate that it is distal colon cancer that is related to endogenous hormonal risk factor of parity. For all cases combined, the present study observed an odds ratio for number of full-term pregnancies of 1.19 (95% CI = 1.00–1.42) for the distal colon. There was, however, no significant heterogeneity in the odds ratios ($P$ = 0.803).

The present study showed an intriguing finding concerning the dissociation of both age at first full-term pregnancy and age at first pregnancy, with subsite-specific risk of colon cancer; the observed increase in odds ratio was found to be significant in distal colon cancer (OR = 1.75), but not significant in proximal colon cancer (OR = 1.17). Potter and McMichael (1983) observed that the risk of cancer in both sides of the colon influenced by age at first live birth. A Norwegian cohort study (Kvale and Heuch), 1991, however, showed that relative risk (RR) of colon cancer related to age at first birth of over 35 was higher on the left side than on the right; the findings were RR for right colon = 0.64, RR for transverse and descending colon = 1.25, RR for sigmoid and rectosigmoid colon = 2.21, and are quite relevant to those of the present study. A similar finding has been reported in another Norwegian birth cohort (Kravdal et al, 1993).

The current, a hospital-based study, is internally valid because the cases and controls are selected from the same source population since the study population was the first-visit outpatient adults at the hospital. The representativeness of cases and controls has been described elsewhere (Yoo et al, 1992; Hirose et al, 1995). The fact that these results are unlikely to be affected by selection bias have been described elsewhere (Inoue et al, 1995). The major limitation of the present study is that the number of cases compared to controls was too small to ensure comparability. It is true that efficiency does not increase appreciably above a control: case ratio of 4:1. However, given a fixed number of cases, a higher control: case ratio certainly does not hurt efficiency, and does lead to a marginal improvement. Thus, we saw no reason not to include the entire control group in the analysis, as opposed to selecting a random sample of controls. It should be reassuring, however, that with regard to comparability, the methodological issue of using all available non-cancer individuals as a control group (Hamajima et al, 1994), as well as the disease profile of cancer-free controls in the present study, have already been discussed elsewhere (Yoo et al, 1992; Hirose et al, 1995; Inoue et al, 1995).

On the basis of a trend analysis, using Japanese mortality statistics for the period 1969–1981, Tajima et al (1985) reported that the age-adjusted death rate for distal colon cancer has fundamentally increased in both genders and, in particular, is higher in the male. The South Australian Cancer Registry, however, reported that, for the period 1977–1980, female rates for distal colon cancer incidence before the age of menopause (approximately age 50) exceeded male rates for that age group (McMichael and Potter, 1983). Slattery et al (1995) observed that, according to the Utah Population Database, the majority of tumours among women diagnosed at age 64 or less were in the distal segment of the colon, while among women 65 or older, the majority of tumours were proximal. Differences in the age-specific incidence of subsite-specific tumours among various countries might influence the results of subsite analysis.

This study confirms that, in Japan, there is a strong association between female reproductive factors and the risk of colorectal cancer. In the comparison of risk factors by colonic subsite, particularly noteworthy was the fact that the significance of age at menarche, of age at first pregnancy and of age at first full-term pregnancy was found to be greater in case of distal colon cancer, with statistical significance. Based on these findings, it can be speculated that hormonal reproductive factors may be closely related to the risk of distal colon cancer, which suggests that through a mechanism not yet understood, sex hormones play an aetiologic role in the carcinogenesis of colon cancer. The recent increase in the incidence of distal colon cancer seen in Japan might be attributed to high consumption of Western-style foods (Inoue et al, 1995), in combination with subsequent changes in reproductive behaviour in younger Japanese women.

### ACKNOWLEDGEMENTS

This study was supported by the Basic Medical Research Fund, Korean Ministry of Education (1995 and 1996). It was also supported in part by a Grant-in-Aid for Cancer Research from the Ministry of Health and Welfare, Japan. The authors thank Drs Robert Dubrow and Harvey Risch for their comments and statistical consultation, and Hiroko Fujikura, Yukiko Yamauchi, Etsuko Nakamura and Takako Kuno for their assistance in data collection.

### REFERENCES

Armstrong B and Doll R (1975) Environmental factors and cancer incidence and mortality in different countries, with special reference to dietary practices. *Int J Cancer* **15**: 617–631

Bostick RM, Potter JD, Kushi LH, Sellers TA, Steinmetz KA, McKenzie DR, Gapstur SM and Folsom AR (1994) Sugar, meat, and fat intake, and non-dietary risk factors for colon cancer incidence in Iowa women (United States). *Cancer Causes Control* **5**: 38–52

Breslow NE and Day NE (1980) *Statistical Methods in Cancer Research, Vol. 1. The Analysis of Case-control Studies*. IARC scientific publication no. 32. International Agency for Research on Cancer: Lyon

© Cancer Research Campaign 1999 

Cantor KP, Lynch CF and Johnson D (1993) Reproductive factors and risk of brain, colon, and other malignancies in Iowa (United States). *Cancer Causes Control* **4**: 505–511

Correa P and Haenszel W (1978) The epidemiology of large-bowel cancer. *Adv Cancer* **26**: 1–141

Cummings JH (1983) Fermentation in the human large intestine: evidence and implications for health. *Lancet* **1**: 1206–1209

de Verdier MG and London S (1992) Reproductive factors, exogenous female hormones, and colorectal cancer by subsite. *Cancer Causes Control* **3**: 355–360

Fox J (1984) *Linear Statistical Models and Related Methods with Applications to Social Research*. John Wiley: New York

Fraumeni JF Jr, Lloyd W, Smith EM and Wagoner JK (1969) Cancer mortality among nuns: role of marital status in etiology of neoplastic disease in women. *J Natl Cancer Inst* **42**: 455–468

Hamajima N, Hirose K, Inoue M, Takezaki T, Kuroishi T and Tajima K (1994) Case-control studies: matched controls or all available controls? *J Clin Epidemiol* **47**: 971–975

Hirose K, Tajima K, Hamajima N, Inoue M, Takezaki T, Kuroishi T, Yoshida M and Tokudome S (1995) A large-scale, hospital-based case-control study of risk factors of breast cancer according to menopausal status. *Jpn J Cancer Res* **86**: 146–154

Howe GR, Craib KJP and Miller AB (1985) Age at first pregnancy and risk of colorectal cancer: a case-control study. *J Natl Cancer Inst* **74**: 1155–1159

Inoue M, Tajima K, Hirose K, Hamajima N, Takezaki T, Hirai T, Kato T and Ohno Y (1995) Subsite-specific risk factors for colorectal cancer: a hospital-based case-control study in Japan. *Cancer Causes Control* **6**: 14–22

Jacobs EJ, White E and Weiss NS (1994) Exogenous hormones, reproductive history, and colon cancer (Seattle, Washington, USA). *Cancer Causes Control* **5**: 359–366

Kampman E, Potter JD, Slattery ML, Caan BJ and Edwards S (1997) Hormonal replacement therapy, reproductive history, and colon cancer: a multicenter, case-control study in the United States. *Cancer Causes Control* **8**: 146–158

Kravdal O, Glattre E, Kvale G and Tretli S (1993) A subsite-specific analysis of the relationship between colorectal cancer and parity in complete male and female Norwegian birth cohorts. *Int J Cancer* **53**: 56–61

Kune GA, Kune S and Watson LF (1989) Children, age at first birth, and colorectal cancer risk. *Am J Epidemiol* **129**: 533–542

Kvale G and Heuch I (1991) Is the incidence of colorectal cancer related to reproduction? A prospective study of 63 000 women. *Int J Cancer* **47**: 390–395

McMichael AJ and Potter JD (1980) Reproduction, endogenous and exogenous sex hormones, and colon cancer. A review and hypothesis. *J Natl Cancer Inst* **65**: 1201–1207

McMichael AJ and Potter JD (1983) Do intrinsic sex differences in lower alimentary tract physiology influence the sex-specific risks of bowel cancer and other biliary and intestinal disease? *Am J Epidemiol* **118**: 620–627

McMichael AJ and Potter JD (1985) Host factors in carcinogenesis: certain bile-acid metabolic profiles that selectively increase the risk of proximal colon cancer. *J Natl Cancer Inst* **75**: 185–191

Martinez ME, Grodstein F, Giovannucci E, Colditz GA, Speizer FE, Hennekens C, Rosner B, Willett WC and Stampfer MJ (1997) A prospective study of reproductive factors, oral contraceptive use, and risk of colorectal cancer. *Cancer Epidemiol Biomarkers Prev* **6**: 1–5

Peters RK, Pike MC, Chang WWL and Mack TM (1990) Reproductive factors and colon cancers. *Br J Cancer* **61**: 741–748

Potter JD and McMichael AJ (1983) Large bowel cancer in women in relation to reproductive and hormonal factors: a case-control study. *J Natl Cancer Inst* **71**: 703–709

Potter JD, Slattery ML, Bostick RM and Gapstur SM (1993) Colon cancer: A review of the epidemiology. *Epidemiol Rev* **15**: 499–545

Robbins SL (1974) *Pathologic Basis of Disease*, pp. 968–969. Saunders: Philadelphia

Weiss NS, Daling JR and Chow WH (1981) Incidence of cancer of the large bowel in women in relation to reproductive and hormonal factors. *J Natl Cancer Inst* **67**: 57–60

SAS Institute Inc (1987) *SAS/STAT Guide for Personal Computers*, Version 6 ed., SAS Institute Inc.: Cary, North Carolina

Slattery ML, Mineau GP and Kerber RA (1995) Reproductive factors and colon cancer: the influence of age, tumor site, and family history on risk (Utah, United States). *Cancer Causes Control* **6**: 332–338

Statistics and Epidemiology Research Corporation (1990) *EGRET*. SERC Inc.: Seattle, Washington

Tajima K, Hirose K, Nakagawa N, Kuroishi T and Tominaga S (1985) Urban–rural difference in the trend of colorectal cancer mortality with special reference to the subsites of colon cancer in Japan. *Jpn J Cancer Res* **76**: 717–728

Yoo KY, Tajima K, Kuroishi T, Hirose K, Yoshida M, Miura S and Murai H (1992) Independent protective effect of lactation against breast cancer: a case-control study in Japan. *Am J Epidemiol* **135**: 726–733

Yoo KY, Tajima K, Miura S, Takeuchi T, Hirose K, Risch H and Dubrow R (1997) Breast-cancer risk factors according to combined estrogen and progesterone receptor status: a case-control analysis. *Am J Epidemiol* **145**: 307–314

Zaridze DG (1983) Environmental etiology of large-bowel cancer. *J Natl Cancer Inst* **70**: 389–400

*© Cancer Research Campaign 1999*
